# Microarray Generation of Thousand-Member Oligonucleotide Libraries

**DOI:** 10.1371/journal.pone.0024906

**Published:** 2011-09-23

**Authors:** Nina Svensen, Juan José Díaz-Mochón, Mark Bradley

**Affiliations:** School of Chemistry, University of Edinburgh, Edinburgh, United Kingdom; Texas A & M University, United States of America

## Abstract

The ability to efficiently and economically generate libraries of defined pieces of DNA would have a myriad of applications, not least in the area of defined or directed sequencing and synthetic biology, but also in applications associated with encoding and tagging. In this manuscript DNA microarrays were used to allow the linear amplification of immobilized DNA sequences from the array followed by PCR amplification. Arrays of increasing sophistication (1, 10, 3,875, 10,000 defined sequences) were used to validate the process, with sequences verified by selective hybridization to a complementary DNA microarray and DNA sequencing, which demonstrated a PCR error rate of 9.7×10^−3^/site/duplication. This technique offers an economical and efficient way of producing specific DNA libraries of hundreds to thousands of members with the DNA-arrays being used as “factories” allowing specific DNA oligonucleotide pools to be generated. We also found substantial variance observed between the sequence frequencies found via Solexa sequencing and microarray analysis, highlighting the care needed in the interpretation of profiling data.

## Introduction

The ability to efficiently and economically generate libraries of defined pieces of DNA would have a myriad of applications, not least in the area of defined or directed sequencing and synthetic biology but also in applications associated with encoding and tagging. There are many examples of where DNA has been used as an encoding device for peptides or small molecules, enabling the high-throughput screening of peptide/small molecule interactions with a range of biological targets [1]–[10].

Perhaps the first use of DNA encoding in this scenario was in the early days of combinatorial chemistry, with bead-based, DNA-encoded libraries composed of up to 800,000 heptapeptides [2]–[3]. This initial approach has since evolved [5], with recent examples of DNA-encoded libraries reported by Nuevolution [7] and Praecis [8] with the synthesis of million to billion member libraries encoded by double stranded DNA [9]. DNA encoded, self-assembled chemical (ESAC) libraries have also been reported [6], with small molecule-linked DNA oligonucleotides combining to give DNA-duplexes encoding two compounds leading to “combination” libraries that can be screened against biological targets.

Another application of DNA libraries is nucleic acid aptamers, which are able to bind molecular targets such as small molecules, proteins, nucleic acids, and even cells, tissues and organisms [11]–[15]. An additional technology that relies heavily on DNA libraries is protein engineering, whereby gene libraries are used to generate libraries of proteins with modified or improved characteristics [16]. This technique has been successfully applied in the areas of modifying enzyme selectivity, altering ligand binding or improving protein stability [17]–[19].

DNA microarrays can be efficiently and economically custom synthesized to contain high numbers (up to millions) of relatively long (up to 200 bp) DNA oligonucleotides [20]. DNA microarrays are typically prepared by: *in-situ* DNA synthesis either by photolithography, where masks (real or virtual) are applied to direct oligonucleotide synthesis [21]–[22]; by inkjet printing mediated synthesis [23]–[25]; or by semiconductor directed synthesis, where an array of individually controlled microelectrodes embedded in a fluidic chamber selectively generate active sites by means of an electrochemical reaction [26]. The attachment of pre-synthesized DNA onto a surface, such as a bead or a glass surface is more expensive and laborious than *in-situ* DNA array synthesis [27].

Efforts have been made to obtain oligonucleotide libraries from a microarray by cleaving the oligonucleotides off the array followed by PCR amplification, thereby generating multiplex DNA libraries for parallel genomic assays [28]. However, this technique is sacrificial, offering no means of reuse of the DNA array. Other examples of the fabrication of DNA libraries include “PCR” on solid supported primers [29] where primers are covalently attached to microarrays with hybridization of specific DNA targets and elongation of the primers generating microarrays of supported DNA libraries with high density of oligonucleotides of any length [30]. This technique has been shown to reduce the undesired, non-selective amplification of DNA oligonucleotides and thereby enhance identification of diagnostic targets [31] and improving SNP detection [32].

**Figure 1:**
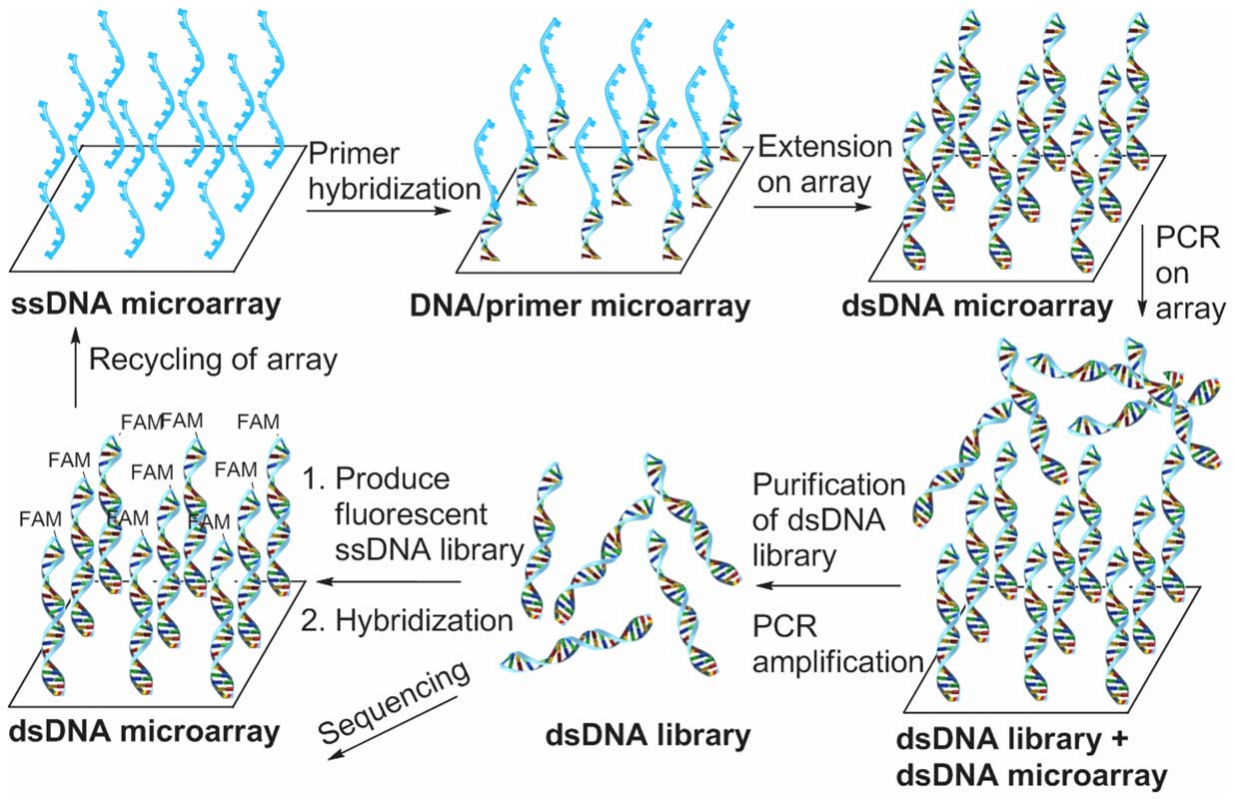
The generation of DNA templates from microarrays and parallel analysis. A ssDNA microarray was incubated with a primer (16 h) followed by elongation using *Taq* polymerase (16 h) producing as dsDNA microarray. The newly synthesized DNA strands were used as templates for solution phase PCR carried out over the microarray leading to amplification of the ssDNA displayed on the microarray. The dsDNA was amplified by PCR to produce fluorescently labeled ssDNA analogous to the ssDNA printed on the microarray. The fluorescently labeled ssDNA was hybridized to a complementary microarray *or* submitted to Solexa sequencing to allow decoding of the amplified ssDNA. FAM = 5(6)-carboxyfluorescein.

Here we demonstrate an approach to the generation of DNA libraries from DNA microarrays allowing the efficient and inexpensive production of custom made thousand-member DNA libraries. The DNA libraries were generated while keeping the array intact and useable for subsequent applications, such as additional rounds of DNA production. This was achieved by fabricating arrays up to 10,000 oligonucleotides followed by “read-off” from the array using a DNA polymerase with subsequent amplification by PCR (Fig. 1). We also show the substantial variance observed in Solexa sequencing compared to conventional microarray analysis.

## Results

### Microarray design

In order to explore the fidelity of the approach, microarrays were designed to contain an increasing number of different DNA oligonucleotides (1, 10, 3,875, or 10,000) and were based on the 17 bp sequences (with a 12 bp variable region) complementary to a previously reported 10,000-member PNA-encoded peptide library [33].

The first oligonucleotide array was designed to contain just one sequence (Table 1), which included domains complementary to primer-1 and primer-2 (Table 1), in a 10×10 pattern. The 10-member oligonucleotide array was designed with the variable domain (12 bp; Table 1, bold region) flanked by domains complementary to primer-1 and primer-2 (Table 1). The oligonucleotides were randomly organized with 4,000 replicates in 4×44,000 sub-arrays. In addition, each sub-array included 4,000 non-complementary DNA oligonucleotides as negative controls.

**Table 1:** General sequences of microarray supported oligonucleotides and primer sequences.

**1-member oligonucleotide microarray**
5′-TCCCAGGGAAAGCATGG**AAGAAGGAGAAC**CTTCTCTCTCTCTCTCTCTCT-3′
**10-member oligonucleotide microarray**
5′-TCCCAGGGAAAGCATGG**HHHHHHHHHHHH**CTTCTCTCTCTCTCTCTCTCT-3′
**3,875 and 10,000-member oligonucleotide microarrays**
5′ *AATGATACGGCGACCACCGAGATCTACACTCTTTCCCTACACGACGCTCTTCCGATCTGG*-
**HHHHHHHHHHHH** *CTTAGATCGGAAGAGCTCGTATGCCGTCTTCTGCTTG-*3′
**Primer-1**
5′-TCCCAGGGAAAGCATGG-3′
**Primer-2**
5′-AGAGAGAGAGAGAGAGAGAAG-3′
**Primer-2-FAM**
5′-FAM-AGAGAGAGAGAGAGAGAGAAG-3′
**Solexa-primer-1**
5′-*AATGATACGGCGACCACCGAGATCTACACTCTTTCCCTACACGACGCTCTTCCGATCT*-3′
**Solexa-primer-2**
5′-*CAAGCAGAAGACGGCATACGAGCTCTTCCGATCT*-3′
**Primer-3**
5′-*CTACACGACGCTCTTCCGATCTGG*-3′
**Primer-4-FAM**
5′-FAM-*GCATACGAGCTCTTCCGATCTAAG*-3′

H = A, C, or T.

The 3,875 and 10,000-member oligonucleotide arrays were designed with the variable domain (12 bp; Table 1, bold region) flanked by domains complementary to Solexa-primer-1 and 2 (Table 1, italic regions) to allow subsequent DNA sequencing. In order to quantitatively assess the amplification of each oligonucleotide on the array the 3,875 oligonucleotide array was designed with scaling of the content of the oligonucleotides with either 1, 2, 4, 8, or 16 replicates of each oligonucleotide in each of the 4×44,000 sub-arrays (Table 2). In addition, each sub-array included 1,375 non-complementary DNA oligonucleotides as negative controls. The 10,000 oligonucleotides were organized randomly with 4 replicates of each in 4×44,000 sub-arrays and each sub-array included 4,000 non-complementary DNA oligonucleotides as negative controls.

**Table 2:** Number of replicates of oligonucleotides on the scaled content 3,875-oligonucleotide microarray.

Number of oligonucleotides**×**number of replicates	Number of spots
2000 oligonucleotides×16:	32,000
1000 oligonucleotides×8:	8,000
500 oligonucleotides×4:	2,000
250 oligonucleotides×2:	500
125 oligonucleotides×1:	125
3,875 oligonucleotides in total:	42,625

### PCR “read-off” microarrays

The first steps in the process involved primer hybridization and elongation on the solid support and required extended reaction times for efficient production of a double stranded (ds) DNA microarray, with one DNA strand covalently attached to the surface. The newly synthesized DNA strands could then function as templates for solution phase PCR carried out over the microarray leading to amplification of the ssDNA displayed on the microarray (Fig. 1).

**Figure 2:**
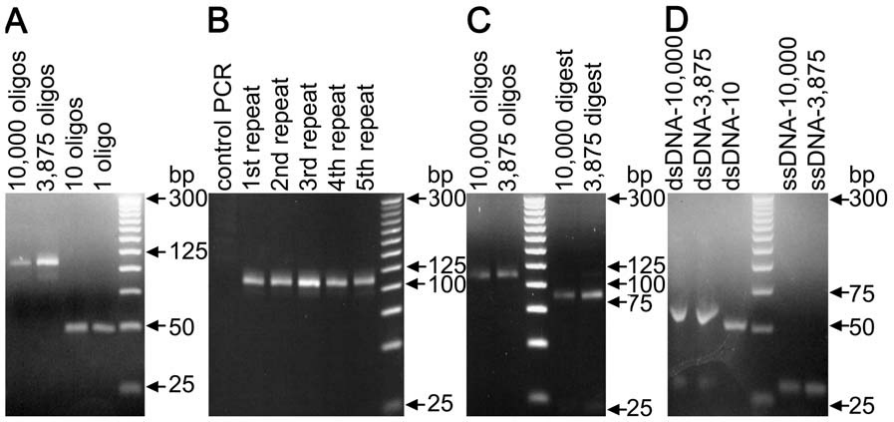
DNA gel electrophoresis. (**a**) PCR products from the 1, 10, 3,875, 10,000 oligonucleotide microarrays. (**b**) Products from 5 repeats of PCR from the 10,000 oligonucleotide array. (**c**) dsDNA-10,000 and dsDNA-3875 (left) and their EcoICRI digestion (right). (**d**) PCR amplification with two primers producing dsDNA-10,000-FAM and dsDNA-3,875-FAM and dsDNA-10-FAM (left) and asymmetric PCR with a single primer producing ssDNA-10,000-FAM and ssDNA-3,875-FAM (right).

PCR “read-off” of the 1-member oligonucleotide array gave a 50 bp band by DNA gel electrophoresis (Fig. 2a). Conventional Sanger sequencing of the PCR amplified product showed the expected oligonucleotide sequence (Table 1). PCR “read-off” of the 10-member oligonucleotide microarray also gave the expected 50 bp band by gel electrophoresis (DNA-10), with the larger 3,875 and 10,000-member oligonucleotide microarrays giving the expected 107 bp bands (DNA-3,875 and DNA-10,000 respectively, Fig. 2a). Furthermore, enzymatic digestion with EcoICRI (recognition sequence: 5′-GAG^▾^CTC-3′) of DNA-3,875 and DNA-10,000 resulted in the two expected fragments (85 bp and 22 bp, Fig. 2c).

Amplification off the 10,000-member oligonucleotide microarray was repeated 5 times after the initial round of primer hybridization, elongation, and washing but without stripping off the newly synthesized DNA and resulted in similar isolated yields of 39–40% (Eq. 1) illustrating that “read-off” can be performed multiple times without damaging the array (Fig. 2b). No product was detected when the “read-off” on the 10,000 oligonucleotide array was carried out without primers (negative control, Fig. 2b).

Previous studies have shown that spacer molecules reduce steric interference of the support on the hybridization efficiency of immobilized oligonucleotides [34], which could also be extended to spacers improving the accessibility of solid supported oligonucleotides for enzymatic reactions. However, this was not an obstacle when using the Agilent arrays, as these include spacers, the nature of which is not disclosed by the manufacturer, that separate the customized 60 bp oligonucleotides from the solid support.

### Microarray hybridization of PCR products

To allow microarray quantification of the DNA microarray “read-off” libraries, these were further amplified by PCR with a FAM-labeled primer and an unlabeled primer (primer-1 and primer-2-FAM for DNA-10, primer-3 and primer-4-FAM for DNA-3,875 and DNA-10,000) producing FAM-labeled dsDNA libraries (DNA-10-FAM, DNA-3,875-FAM, DNA-10,000-FAM; Table 1 and Fig. 2d).

The dsDNA-10-FAM was hybridized onto a complementary DNA microarray identical to the “read-off” DNA microarray (above). Fluorescent microarray imaging in combination with BlueFuse technology (BlueGenome) was used to quantify the intensity of the FAM-label and thereby determine the amount of DNA hybridized to each spot (ArrayExpress: E-MEXP-3102).

The double stranded DNA-3,875 and DNA-10,000 libraries needed to be hybridized to DNA microarrays that encode only the 12 bp variable domain of the DNA-10,000 library (Table 1, bold) arrays contained four replicates of each sequence in the 10,000 member library as well as 4,000 non-complementary DNA oligonucleotides as negative controls. However, hybridization of the 12 bp microarray supported oligonucleotides with a 107 bp dsDNA library is very challenging due to the competition between the non-microarray complementary 107 bp ssDNA strands and the 12 bp microarray supported ssDNA strands. Microarray hybridization used single stranded DNA, which was generated by asymmetric PCR with a single primer (primer-4-FAM, Table 1). This produced microarray complementary ssDNA libraries (ssDNA-3,875-FAM and ssDNA-10,000-FAM, Fig. 1 and 2d), which were hybridized onto microarrays that were complementary to the 12 bp variable domain (Table 1, bold) and the microarrays were imaged as described above.

### Quantification of microarray hybridizations

**Figure 3:**
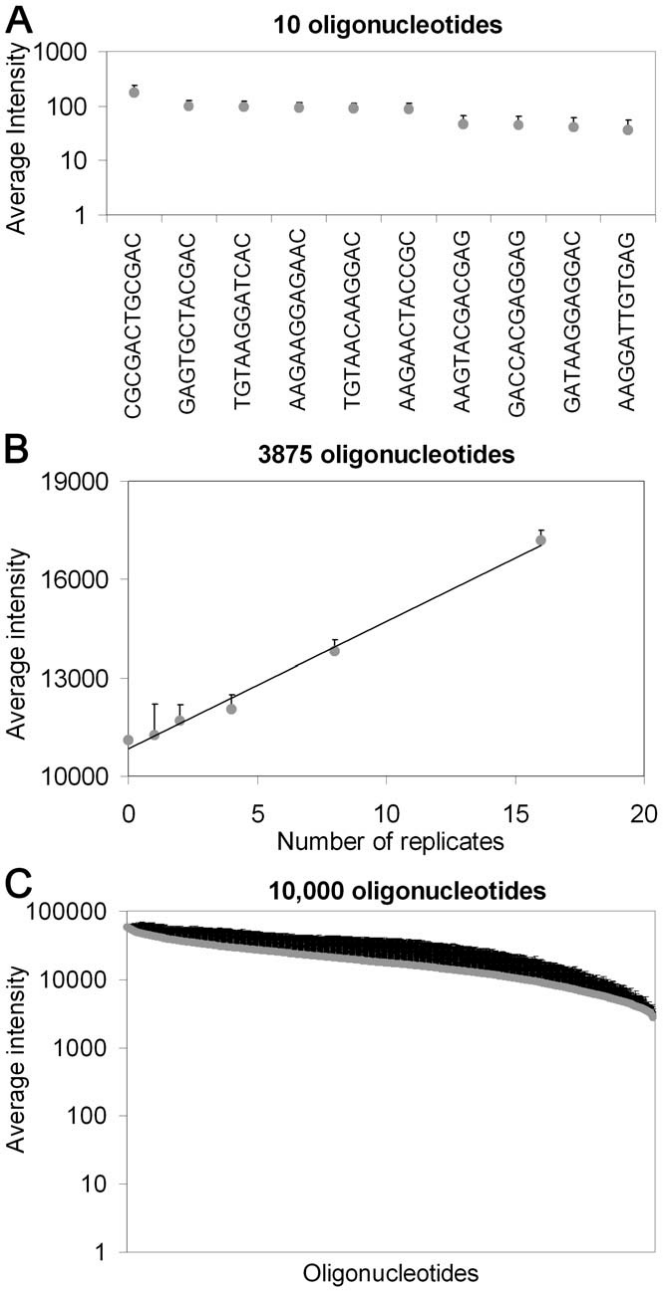
The background corrected average intensities plotted versus the number of replicates. (**a**) The dsDNA-10-FAM library. (**b**) The ssDNA-3,875-FAM library. (**c**) The ssDNA-10,000-FAM library. Error bars indicate ± s.d.

Raw microarray data were obtained from Bluefuse, which allows grid alignment and signal estimation. The top ∼5% and the bottom ∼5% of each of the replicate-sets were removed as outliers (erroneous values caused by dust, scrapes etc. [35]–[36]) and the background corrected average intensity was calculated for all of the replicate sets and for the intensity of the non-coding negative control features on each microarray. In order to assess the efficiency of the microarray “read-off” and subsequent PCR amplification the average microarray intensities were plotted against the number of replicates (Fig. 3).

The slight differences in average intensities for the 10 oligonucleotide graph (Fig. 3a) arise from differences in the number of copies of each oligonucleotide on the “read-off” array as well as differences in secondary structures and T_m_ of the oligonucleotides as these characteristics greatly affect the hybridization efficiency. Thus, a curved distribution of microarray intensity versus the oligonucleotide sequences is expected [37]. The narrow range of the average intensities and their low standard deviation values in combination with curved distribution of the 10 oligonucleotide graph illustrate that the microarray “read-off” had occurred uniformly over the whole array.

The graph for the 3,875 oligonucleotides shows a linear relationship between the microarray intensities versus the number of replicates illustrating that the 3,875 DNA templates had been “read-off” and amplified relative to the number of replicates of oligonucleotides on the microarray (Fig. 3b). Each data point in the 3,875 oligonucleotides graph represents the average of many different oligonucleotides (Table 2), each with different synthesis efficiencies, T_m_, and secondary structures. Consequently, the effects of these parameters on the hybridization efficiency cancelled each other out when the average intensity was calculated over many different sequences. This resulted in a smoother distribution of the 3,875 oligonucleotide graph compared to that of the 10 oligonucleotide graph.

The average intensity versus the number of replicates for the 10,000 oligonucleotides showed a curved distribution illustrating that the microarray “read-off” occurs uniformly over the high-content arrays with few replicates of each oligonucleotide (Fig. 3c).

### Illumina Solexa sequencing

Solexa sequencing of the DNA-10,000 oligo-pool identified 9976 sequences from the possible 10,000 DNA oligonucleotides synthesized on the DNA microarray giving a loss rate of 0.2% (24 oligonucleotides not seen out of 10,000, Table 3 and ArrayExpress: E-MTAB-540). Noticeably, the oligonucleotides not seen via sequencing all had one of the following consensus sequences (X = any base): CGC-XXX-XXX-CGC, CGC-XXX-CGC-XXX, CGC-CGC-XXX-XXX, CAC-GAX-XAG-TGC (Table 3).

**Table 3:** Oligonucleotide sequences not seen by Solexa sequencing and their background-corrected average microarray intensities.

Sequence	Microarray intensity
CACGACGAGTGC	1.53 E+04
CACGAGAAGTGC	2.64 E+04
CACGATAAGTGC	1.12 E+04
CACGATGAGTGC	6.38 E+03
CGCCACAAGCGC	1.53 E+04
CGCCACGAGCGC	2.33 E+04
CGCCGCCGCCGC	3.81 E+04
CGCCGCGAGCGC	3.64 E+04
CGCGACGAGCGC	1.69 E+04
CGCGAGCGCCAC	2.06 E+04
CGCGAGGAGCGC	1.80 E+04
CGCGAGGATCGC	2.54 E+04
CGCGATAAGCGC	3.57 E+04
CGCGATGAGCGC	2.37 E+04
CGCTACAAGCGC	3.47 E+04
CGCTACGAGCGC	2.30 E+04
CGCTGCAAGCGC	3.25 E+04
CGCTGCGAGCGC	3.28 E+04
CGCTGTAAGCGC	2.37 E+04
CGCTGTCGCCGC	2.09 E+04
GAGCGCAAGCGC	3.75 E+04
GAGCGCCGCCGC	3.52 E+04
GAGCGCCGCGAC	2.13 E+04
AAGCGCAAGCGC	1.47 E+04

**Figure 4:**
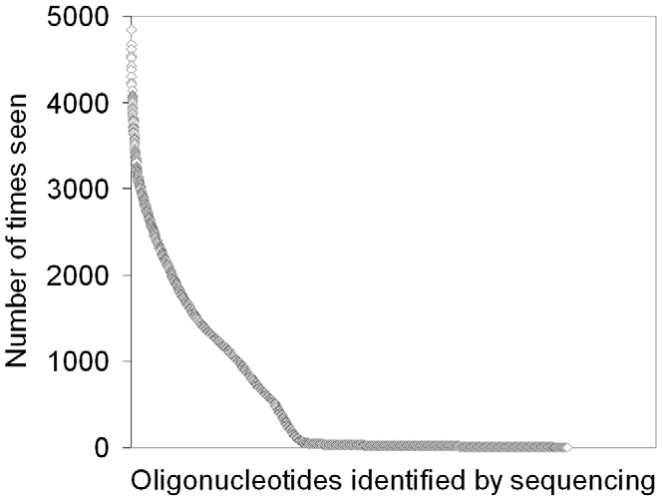
The number of times each oligonucleotide was seen by Solexa sequencing plotted versus the oligonucleotide sequences. 36-bp reads of the Solexa primer of the dsDNA-10,000 oligo-pool generated by “read-off” the 10,000 oligonucleotide microarray.

Of interest was that the 9976 sequences were seen between 1 to 4837 times each (Fig. 4). This significant difference in the number of reads of each oligonucleotide was initially thought to correspond to an unexpected large difference in the actual amount of the respective oligonucleotide in the library. Closer examination of the sequences revealed that the oligonucleotides that had poor frequencies of observation had the same consensus sequences as the non-identified oligonucleotides (Table 3). It is important to note that all of the oligonucleotides not seen by sequencing were observed by microarray hybridization in substantial amounts (the arbitrary microarray intensities where in the range of 6,000–38,000 compared to the full intensity range of 2,700–55,000; Table 3 and ArrayExpress: E-MEXP-3102). Thus, no evidence of low synthesis rate of the high GC-content CGC-codon was observed in the microarray hybridization experiment and the low observation frequency of CGC containing oligonucleotides in Solexa sequencing cannot be explained by low synthesis efficiencies.

The relatively high number of rare hits seen in Fig. 4 may be explained by the high similarity between the oligonucleotides (each oligonulceotide differs from other library members with as little as one nucleotide [33]). This may present difficulties in distinguishing truly different sequences from sequence errors, which prevents exclusion of sequences that arise from changes at conserved positions in the library. Thus, when unique sequences seen with Solexa sequencing were tallied, all unique sequences were counted.

Based on the data from sequencing and the microarray screening it can be assumed that the relative amounts observed by sequencing are an effect of the *actual* amounts of the oligonucleotides in the sample, but that this is secondary to the efficiency of the base calling of the respective sequence. Similarly, significant skewing has previously been reported in Solexa sequencing of a PCR-amplified synthetic oligonucleotide library [38].

### Determination of the PCR error rate

The PCR error rate was calculated using the formula given by Hayes (1965; Materials and Methods, Eq. 2, [39]): [2×observed error number]/[total DNA length examined×effective number of duplications]. The observed error number per sequence was 5.499 (Materials and Methods). However, this observed error number includes the combined errors in both the PCR “read-off” microarray and the subsequent PCR amplification with Solexa-primers.

The effective number of duplications can be calculated from the template-product ratio. The amount of PCR product amplified from ∼2.9×10^−13^ g of microarray supported template DNA (Agilent) was determined to be 111 µg, and the effective number of duplications was calculated to be 18.8 (Materials and Methods). Therefore, the error rate was calculated to be 9.7×10^−3^/site/duplication.

This error rate is slightly higher than the error rate typically observed for the *Taq* polymerase (1.0×10^−4^/site/duplication) [40]. However, considering the error rate of 9.7×10^−3^/site/duplication is the combined error rate for two subsequent PCRs and that the first of these included PCR “read-off” a microarray an increased error rate would be expected compared to literature values.

## Discussion

Four microarrays with 1, 10, 3,875 or 10,000 different oligonucleotide sequences were utilized to determine whether they could be used as a platform for large scale DNA synthesis. A novel microarray “read-off” technology was established that allows high-throughput amplification of microarray supported DNA probes and the production of DNA libraries containing tens of thousands of members.

DNA sequencing and microarray hybridization of 1, 10, 3,875, and 10,000 DNA oligonucleotide “read-off” libraries illustrated that microarray “read-off” had occurred uniformly over the whole of the high-content DNA microarrays, and that the amount of oligonucleotide in the library mixture was determined by the number of replicates of each oligonucleotide on the “read-off” array. The DNA-arrays could be used as “factories” allowing specific DNA oligo pools to be generated with or without masking. The PCR error rate for the combined PCR “read-off” microarray and subsequent PCRs was calculated to be 9.7×10^−3^/site/duplication, which is relative to the error rate typically observed for the *Taq* polymerase (1.0×10^−4^/site/duplication) [40].

This technique offers efficient and inexpensive generation of thousands of defined oligonucleotides, which could allow the rapid synthesis of specific primers for use in genome sequencing and genotyping assays or DNA-encoding methods and aptamer screening. Furthermore, this method gives easy access to unpurified mixtures of microarray-synthesized oligonucleotides, which have been used directly in generation of high-quality gene assembly [41]. This technique could also allow production of defined DNA libraries by employing an appropriate microarray design. For example, a microarray with 100 defined subarrays, each with repeats of a single oligonucleotide, would enable synthesis of separate oligonucleotide pools simply by using a cover-slip with 100 separate chambers [42].

Another application of the technique could be the synthesis of defined siRNA libraries by employing an RNA polymerase [43] rather than DNA polymerase, which would allow pools of siRNA to be synthesized from DNA microarrays [44]–[48]. Again, masking could allow rapid generation of separate oligonucleotide pools and the array to be re-used.

Interestingly we also observed that the comparative results of microarray hybridization analysis did not correlate with those of Solexa sequencing due to specific consensus sequences that sequenced poorly. The oligonucleotides not seen by sequencing were identified in substantial amounts by microarray hybridization. Together with the relatively low PCR error rate of the combined PCR “read-off” microarray and subsequent PCR amplification this demonstrates that the “read-off” approach is not sequence dependent but that the Solexa sequencing is. Similarly, significant skewing has previously been reported in Solexa sequencing of a PCR-amplified synthetic oligonucleotide library [40], perhaps suggesting that comparative mRNA profiling analysis on Solexa needs to be done with care.

## Materials and Methods

### Microarray manufacture

The 1-member oligonucleotide microarray was generated by contact printing a 3′-amino modified DNA oligonucleotide (Microsynth) onto a Codelink® slide in a 10×10 pattern. After printing the unreacted sites on the slide were blocked with ethanolamine and the array was washed briefly with 0.2% SDS in 4× SCC (Fisher Scientific), 0.1% SDS in 2× SCC for 2×5 min, 0.2× SCC for 5 min, and 0.1× SCC for 5 min, and dried under a flow of N_2_. All other DNA microarrays were custom fabricated by Oxford Gene Technologies (OGT).

### DNA gel electrophoresis

Samples (20–30 µL) were prepared with 6× Blue/Orange Loading Dye (5 µL, Promega) and DNA grade H_2_O were run on a 5 (w/v)% agarose gel (Promega Preparative grade for small fragments) in 1× Tris Borate EDTA (pH 8.3, TBE, Fisher Scientific) buffer for approximately 1 h. The gel was analyzed under UV light and the appropriate bands were exercised with a scalpel. DNA was purified using a QIAEX II Agarose Gel Extraction Kit (Qiagen) according to the manufacturer's protocol.

### PCR “read-off” microarrays

Elongation reaction mix (200 µL) without primers was prepared according to a Promega standard protocol using a PCR Master Mix (Promega, 25 U/mL *Taq* Polymerase, 200 µM dNTP, 1.5 mM MgCl_2_) was loaded onto the microarray using an Agilent hybridization cover slide. The first elongation step was carried out at 50°C (primer-2) or 55°C (Solexa-primer-2) for 16 h (overnight). Hereafter, the reaction mixture was removed using a pipette and fresh PCR reaction mix (200 µL; Promega, 25 U/mL *Taq* Polymerase, 200 µM dNTP, 1.5 mM MgCl_2_) with primer-1 and 2 (0.1 µM) *or* Solexa-primer-1 and 2 (0.1 µM) were loaded onto the microarray and a standard PCR cycle was set up in a GeneMachines® Hyb4 automated hybridizer [40 cycles, denaturation at 94°C for 30 s for 10 cycles and 88°C for 30 s for 30 cycles, annealing at 49°C for 1 min, elongation at 50°C for 5 min (primer-1 and 2), *or* denaturation at 94°C for 30 s, annealing at 65°C for 1 min, elongation at 70°C for 1 min (Solexa-primer-1 and 2)]. In addition, an initial 3 min denaturation step at 94°C and a final 15 min elongation step at 50°C (primer-2) or 70°C (Solexa-primer-2) were carried out. Immediately after the PCR had finished the reaction mix was collected using a pipette and the microarray washed with H_2_O (3×50 µL). The aqueous fractions were pooled together and concentrated in a speed-vac followed by purification by preparative DNA gel electrophoresis as described above (dsDNA-10: 0.70 µg, 40% isolated yield, dsDNA-3875: 1.75 µg, 40% isolated yield, dsDNA-10,000: 1.65 µg, 40% isolated yield, Eq. 1).
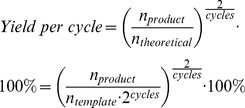
(1)


### PCR in solution

The purified products (250 ng) from each of the PCR “read-off” microarrays were used as templates in another round of PCR with primer-1 and 2 (1 µM) *or* Solexa-primer-1 and 2 (1 µM) in a 1× PCR Master Mix (200 µL; Promega, 25 U/mL *Taq* Polymerase, 200 µM dNTP, 1.5 mM MgCl_2_) in a Techne TC-312 PCR cycler with the same cycle as on the microarray. After PCR the samples were concentrated in a speed-vac followed by purification by preparative DNA gel electrophoresis as described above (dsDNA-3875-2: 1.11 µg, 27% isolated yield, dsDNA-10,000-2: 16.8 µg, 30% isolated yield). This was followed by another round of PCR in solution carried out with DNA (250 ng) with primer-1 and primer-2-FAM (2.5 µM) *or* primer-3 and primer-4-FAM in a 1× PCR Master Mix (200 µL; Promega, 25 U/mL *Taq* Polymerase, 200 µM dNTP, 1.5 mM MgCl_2_) in a Techne TC-312 PCR cycler [2.5 µM, Sigma-Aldrich, 40 cycles, denaturation at 94°C for 30 s for 10 cycles and 88°C for 30 s for 30 cycles, annealing at 49°C for 1 min, elongation at 50°C for 5 min (primer-1 and primer-2-FAM), *or* denaturation at 94°C for 30 s, annealing at 58°C for 1 min, elongation at 66°C for 1 min (primer-3 and primer-4-FAM)]. In addition, an initial 3 min denaturation step at 94°C and a final 15 min elongation step at 50°C (primer-1 and primer-2-FAM) or 66°C (primer-3 and primer-4-FAM) were carried out. After PCR the samples were concentrated in a speed-vac followed by purification by preparative DNA gel electrophoresis as described above (10.5 µg dsDNA-10-FAM, dsDNA-10,000-FAM: 20.5 µg, 29% isolated yield, dsDNA-3875-FAM: 28.3 µg, 30% isolated yield). dsDNA-10,000-FAM and dsDNA-3,875-FAM were used as templates in ssDNA PCR amplification with the FAM-Microarray Primer (10 µM) in 1× PCR Master Mix (600 µL; Promega, 25 U/mL *Taq* Polymerase, 200 µM dNTP, 1.5 mM MgCl_2_) in a Techne TC-312 PCR cycler with the same cycle as described before for this primer. After PCR the samples were concentrated in a speed-vac followed by purification by preparative DNA gel electrophoresis (7.60 µg ssDNA-3875-FAM, 6.04 µg ssDNA-10,000-FAM).

### Digestion Analysis

dsDNA-10.000-2 (200 ng) *or* dsDNA-3875-2 (200 ng) were digested with 0.25 units/µL of EcoCRI (Promega) in 1× RE buffer (20 µL, Promega) containing 0.1 µg/µL Acetylated BSA (Promega) at 37°C for 4 h followed by analytical DNA gel electrophoresis.

### Hybridization of the PCR product

The purified fluorescent PCR constructs were dissolved in 0.1% SDS in 4× SSPE buffer (110 µL; 0.6 M NaCl, 40 mM NaH_2_PO_4_, 5 mM EDTA in H_2_O at pH 7.4) and denatured at 65°C for minimum 5 min. This solution was hybridized on a customized DNA array (OGT) in an Agilent hybridization chamber from 65–27°C over 24 h (conditions were optimized for exclusion of mismatches during hybridization). The arrays were washed with 0.2% Sodium Dodecyl Sulphate (SDS, Promega) in 2× Saline-Sodium Citrate (SSC, 20 mL, Promega) for 5 min, 0.2× SSC (20 mL) for 5 min, 0.1× SSC (20 mL) for 5 min, and briefly rinsed in DNA grade H_2_O (20 mL) and Tris buffer at pH 8.0 (20 mL, 10 mM) and dried under a N_2_ flow. The microarrays were imaged with a Tecan LS Reloaded microarray scanner using a FITC filter and the images were analyzed using Bluefuse (BlueGenome) software (ArrayExpress, accession number E-MEXP-3102, all microarray data complies with the Minimal Information About a Microarray Experiment (MIAME) guidelines.).

### Illumina Solexa sequencing

dsDNA-LL10,000 (200 nmol) was Illumina sequenced with 36-base reads off the Solexa-primer-1 domain at the end of each oligonucleotide (The GenePool, The University of Edinburgh). The resulting reads were clustered against a list of the 10,000 oligonucleotides in the 10,000 library and a list of the identified sequences was generated including the number of times each oligonucleotide was seen. Another list of the sequences not seen by Illumina Solexa sequencing was generated (ArrayExpress, accession number E-MTAB-540).

### PCR error rate calculations

The PCR error rate was estimated using the formula given by Hayes (1965, Eq. 2):
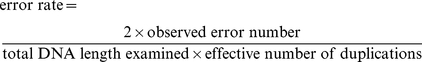
(2)


The observed error number per sequence was calculated as follows:
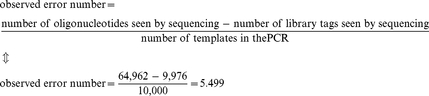
(3)


The length of the microarray supported DNA templates is 60 bp (see table 1) and the approximate amount of DNA template on the 10,000 member array (m) is calculated from Eq. 4–5 based on the manufacturer's specifications of the number of molecules per spot:
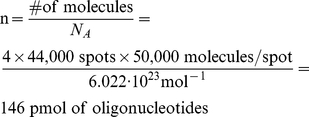
(4)

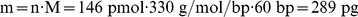
(5)


After elongation on the microarray and PCR the product (1.65 µg; dsDNA-10,000) was used as template in a subsequent PCR with Solexa primers. The amount of PCR product obtained (dsDNA-10,000-2) was 110.8 µg. The effective number of duplications (# of cycles) was calculated from Eq. 6:
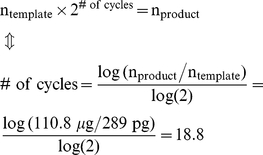
(6)Thus, the error rate was calculated from Eq. 2 to be 9.7*10^−3^/site/duplication.
